# Prevalence of Multiple Drug-Resistant Bacteria in the Main Campus Wastewater Treatment Plant of Wolaita Sodo University, Southern Ethiopia

**DOI:** 10.1155/2022/1781518

**Published:** 2022-11-23

**Authors:** Chimdesa Adugna, Krishna Moorthy Sivalingam

**Affiliations:** Department of Biology, College of Natural and Computational Sciences, Wolaita Sodo University, P.O. Box 138, Wolaita Sodo, Ethiopia

## Abstract

Wastewater treatment plants (WWTPs) are important reservoirs for the development of drug resistance and a potential route for the dissemination of antibiotic resistance genes (ARGs) in the environment. One of the most serious challenges in Ethiopia is the widespread emergence of antibiotic resistance among bacterial pathogens. The bacteria were isolated between September 2018 and May 2019 from the main campus of Wolaita Sodo University in Southern Ethiopia. Using an enrichment process and selective media isolation, 380 wastewater treatment plant samples were collected and screened for the presence of various bacterial isolates. Of a total of 380 wastewater treatment samples, 136 were isolated. Positive prevalence was documented in 136 sample isolates of bacteria from six genera. *Escherichia coli* 34 (8.94%), *Salmonella* spp. 15 (3.94%), *Shigella* spp. 32 (8.42%), *Staphylococcus aureus* 23 (6.05%), *Pseudomonas aeruginosa* 21 (5.52%), and *Proteus* spp. 11 (2.89%). The general prevalence of bacterial isolates was assessed, and 136 (37.58%) samples tested positive for culture. Furthermore, isolates were used to determine sensitivity/resistance patterns using the Kirby–Bauer disc diffusion method and the agar well diffusion technique, respectively. Multiple drug resistance isolates and multiple values of the antibiotic resistance index were evaluated and recorded according to the resistant pattern. Some organisms were sensitive to sparfloxacin and tobramycin, while *Staphylococcus aureus* was sensitive to methicillin and others showed the highest resistance. At least four of the seven antibiotic classes were found to be resistant to multiple drug resistance isolates, and some classes of antibiotics were found to be highly sensitive to these isolates. Multiple antibiotic resistance index values ranged from 0.37 to 0.75, with *Salmonella* spp., *Shigella* spp., and *Staphylococcus aureus* having the highest score values. The current study has shown that some of the bacterial isolates were resistant to common antibiotics. Therefore, it is recommended that the emergence of multiple drug resistance increased rapidly, pathogenic bacteria inappropriate treated wastewater treatment plant systems were continuously contaminated, and bacterial resistance increased day by day as a result of environmental factors. As a result, due to the serious challenges facing the community's health, multiple drug-resistant prevention and control strategies must be implemented.

## 1. Introduction

### 1.1. Background of the Study

Previously, wastewater treatment plants were characterized as “hotspots” for large quantities of antibiotic-resistant bacteria (ARB) and antibiotic resistance genes (ARG) [1]. According to a news release from the Canadian government, “wastewater treatment plants represent an important control point in the actions taken to minimize the development of antibiotic resistance” [2]. The prevalence, distribution, and transfer of AR and resistance genes among bacterial populations within municipal wastewater treatment plant (WWTP) systems have received little study. These data are critical for identifying public health hazards and treatment alternatives. In 2000, the World Health Organization (WHO) issued a study identifying antibiotic resistance (AR) as one of the most pressing human health concerns of the twenty-first century, calling for a “global plan to contain resistance” [3]. Some resistant microorganisms detected in clinical settings may have obtained their resistant genes from environmental reservoirs [4]. As a result, the link between the environment and clinical resistance has become more of a concern, and the discovery and management of resistant reservoirs are of interest.

It is still debatable whether wastewater treatment processes eliminate resistance elements or potentially enrich them. By examining the prevalence of ARB and ARGs in different WWTP systems and examining the effects of different treatment steps, we can advance our understanding of their role in the dissemination of resistance elements into the environment. This may help to highlight the severity of treatment plants' role in the increase of AR, as well as viable solutions to reduce their contribution to the problem. WWTPs discharge their wastewater into receiving habitats such as rivers, lakes, or seas after treatment. Currently, there are no laws or recommendations that govern the maximum levels of ARB, ARG, or antibiotics (AB) that can be released into the receiving environment. As a result, AR determinants remaining after therapy end up in the environment and are not monitored. Once in the environment, genetic elements such as ARGs can move among bacteria and be acquired by hazardous diseases [5]. Antibiotics can also act as a selective factor in the preservation and acquisition of ARGs, increasing AR even more in receptive environments [6]. Other anthropogenic activities, in addition to WWTP effluents, can increase ARGs in the environment, with higher levels related to areas closer to urbanization and agriculture [7, 8].

Runoff from these places, as well as the discharge of WWTP effluents into watersheds, increases the mobility of AR determinants, allowing them to proliferate throughout the environment [5]. As previously stated, there are currently no laws governing what constitutes large amounts of ARG in wastewater or environmental regions such as surface waterways [9]. Investigating a combination of ARG levels in wastewater, effluent receiving environments, and other surface waters can thus provide a starting point for defining what levels are actually high as well as assessing the influence of different sources (e.g., WWTP effluents, proximity to urbanization, and agriculture) on the increasing AR problem. The spread of antibiotic-resistant bacteria is a major public health concern, and it is well recognized that aquatic habitats serve as reservoirs for antibiotic-resistant bacteria [5, 10, 11]. Municipal wastewaters provide an ideal habitat for the development and interchange of genetic material between strains, making them key reservoirs of antibiotic-resistant bacteria. Although wastewaters are treated to minimize the bacterial burden before being released into the environment, a tiny amount of resistant bacteria still reach the natural environment. The primary goal of this study was to determine the prevalence of multiple drug-resistant bacteria in the wastewater treatment plant (WWTP) on the main campus of Wolaita Sodo University, Southern Ethiopia.

### 1.2. Statement of the Problems

The spread of MDR among pathogenic and commensal bacteria is a global health concern. Each year, about 17 million people die from infectious diseases around the world, most of which are caused by bacteria. The Centers for Disease Control and Prevention [12] Untreated wastewater treatment or inappropriately treated wastewater plants are a serious issue in our country because many universities and hospitals release waste water into rivers and streams. This wastewater contains many pathogenic bacteria that are resistant to multiple drugs, which is the worst problem for public health and the most expensive to treat.

## 2. Materials and Methods

### 2.1. Description of the Study Area

**Figure 1:**
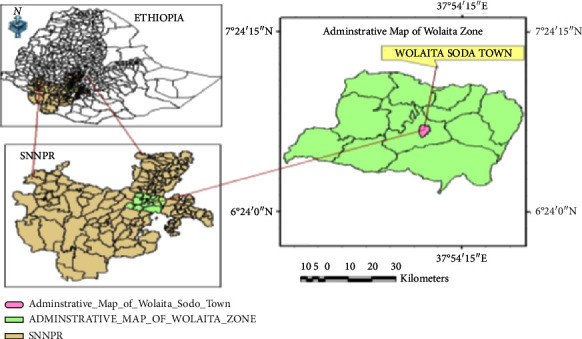
Map of the study area.

The research was carried out on the campus of Wolaita Sodo University in Southern Ethiopia between September 2018 and May 2019. The research region is located 320 kilometers south of Addis Ababa, Ethiopia's capital. Its elevation ranges from 1,650 to 2,980 meters above sea level, and its annual average temperature is 25–35°C. The Wolaita zone is located at the edge of East Africa's Great Rift Valley, between 70 north latitude and 37° 45 east longitude. Wolaita Sodo Town is one of the region's fastest-growing communities. It is in the heart of the Southern Regional State of Nations, Nationalities, and Peoples. According to CSA forecasts, the total population of the town was expected to be 1,02,922 in 2012, with 54,315 males and 48,607 females, with an annual population growth rate of roughly 5.3%, as shown in Figure 1.

### 2.2. Sampling Techniques and Sample Collection

The current investigation was carried out between September 2018 and May 2019 to investigate the prevalence of multiple drug-resistant bacteria in the wastewater treatment plant at the Wolaita Sodo University campus, Southern Ethiopia. A total of 380 wastewater treatment samples were collected at various phases of the wastewater treatment process. Wastewater samples were collected twice a week at two time points a day (10 : 30 am and 2 : 30 pm) from each of the following stages: primary stage, secondary stage, and tertiary stage. The samples were collected in 100 ml sterile plastic bottles and stored in an ice box, and each sample was appropriately labelled. Finally, the samples were delivered to the Department of Biology's post-graduate microbiology laboratory on the campus of Wolaita Sodo University.

### 2.3. Maintenance and Preservation of Culture Strains

The organisms were grown in suitable medium for 24 hours before being preserved in a nutrient agar slant at 2–8°C in a refrigerator, and the cultures were used for routine laboratory work within two weeks. Strains were preserved in brain heart infusion broth (BHIB) (HiMedia-LQ210D) with 20% glycerol and stored frozen at −20 C without noticeable loss of viability until further research [13].

### 2.4. Antimicrobial Susceptibility Test

The disc diffusion method was used to examine the sensitivity and resistance pattern of wastewater treatment sample isolates [14]. Culture plates were made by putting 20 ml of Mueller–Hinton agar (MHA) on a plate (HiMedia-M173). Bacterial isolates were examined for the presence of the following regularly prescribed drugs: imipenem (10 *μ*g), meropenem (10 *μ*g), ertapenem (10 *μ*g), gentamycin (10 *μ*g), amikacin (30 *μ*g), amoxicillin-clavulanic acid (30 *μ*g), aztreonam (30 *μ*g), nalidixic acid (30 *μ*g), sparfloxacin (5 *μ*g), ofloxacin (5 *μ*g), norfloxacin (10 *μ*g), nitrofurantoin (300 *μ*g), ceftriaxone (30 *μ*g), ciprofloxacin (5 *μ*g), tobramycin (10 *μ*g), methicillin (5 *μ*g), vancomycin (5 *μ*g), tetracycline (30 *μ*g), co-trimoxazole (25 *μ*g), amikacin (10 *μ*g), ampicillin (10 *μ*), penicillin (10 *μ*g), chloramphenicol (30 *μ*g), doxycycline (30 *μ*g), and cefoxitin (30 *μ*g). Zones of inhibition were measured and compared with the National Committee for Clinical Laboratory Standards (NCCLS) guidelines [15].

### 2.5. Criteria for the Selection of Multiple Drug-Resistant (MDR) Strains

MDR strains were defined as bacteria isolates that were resistant to three or more antibiotics from various structural classes [16]. In the current investigation, antibiotics from various classes were used, and a maximum of eight antibiotics were used against isolates from each genus.

### 2.6. Indexing of Multiple Antibiotic Resistance (MAR) of Isolates

When applied to a single isolate, the MAR index is defined as *a*/*b*, where *a* is the number of antibiotics to which the isolate was resistant and *b* is the number of antibiotics to which the isolate was exposed [17, 18]. For example, if the isolate was treated with ten antibiotics and was resistant to five of them, its index would be 5/10, or 0.5. A MAR index score greater than 0.2 is thought to have come from high-risk sources of contamination, such as humans, commercial poultry farms, pigs, and dairy cattle, where antibiotics are often used.

#### 2.6.1. Data Entry and Analysis

The collected data were validated, entered, and stored in a Microsoft Excel® spreadsheet, which was also used to calculate means and proportions. After the completion of the AST, each antibiotic measurement was recorded using the standard chart, such as sensitivity, intermediate, and resistance. Furthermore, based on AST, the percentage of sensitivity/resistance was calculated, multidrug-resistant bacteria (MDR) were evaluated, the multiple antibiotic-resistant index (MAR index) was calculated, and findings were reported in appropriate tables and figures.

#### 2.6.2. Ethical Considerations

The study was approved by the Ethical Review Board/Committee of Wolaita Sodo University, and permission was obtained to conduct the research as far as objectives and methodologies were concerned. An ethical approval statement was collected with an ethical approval number (Ref. WSU 41/31/361). The results of the study were communicated to the responsible bodies for beneficiary measures. Authors and Institutional Review Committee/Board of WSU confirmed that this study was able to conduct without clinical trial registration number due to Ethiopian education policy and this not allowed.

## 3. Results

A total of 380 different samples were collected aseptically from the wastewater treatment plant on the main campus of Wolaita Sodo University. The SPC method was used to count bacteria in all 380 samples, and 136 bacterial isolates were found to be positive. Bergey's Manual of Systematic Bacteria was used to identify isolated bacterial strains. MacConkey agar, selective medium, and nutrient broth were used to enrich all positive SPC samples. After inoculation, the enriched samples were aseptically inoculated in various selective media and screened for bacteria isolates.

### 3.1. Standard Plate Count

**Figure 2:**
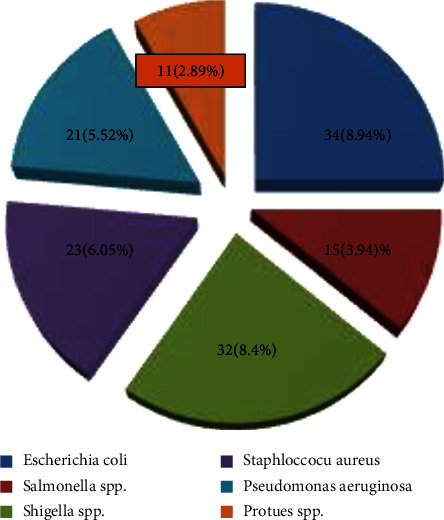
Overall prevalence of bacterial isolates from wastewater treatment plants.

A positive result indicated a colon count greater than 100 cfu/ml. Normally, a standard plate count was used to determine the composition of water and wastewater samples. Among the 380 wastewater samples, 136 were positive (having more than 100 cfu/ml in the wastewater sample considered positive), while the remaining samples were negative using the SPC method. Enrichment and selective media isolation were performed in all 136 samples. The overall prevalence of bacterial isolates was assessed and recorded. Of the 380 samples screened, 136 (35.78%) were culture-positive.  Figure 2 shows that among the culture-positives, six predominant genes were isolated, such as *Escherichia coli* 34 (8.94%), *Salmonella* spp. 15 (3.94%), *Shigella* spp. 32 (8.42%), *Staphylococcus aureus* 23 (6.05%), *Pseudomonas aeruginosa* 21 (5.52%), and *Proteus* spp. 11 (2.89%). Furthermore, the Kirby-Bauer disc diffusion method was used to test all 136 isolates for sensitivity/resistance patterns. Finally, an isolates that were resistant to three or more kinds of antibiotics was classified as a multidrug-resistant organism. Tables 1–7 include the results, which were recorded and collated as shown in Figure 2.

The percentage of sensitivity/resistance of several antibiotic classes against *Escherichia coli* was determined. Four classes of eight antibiotics were used against 34 *E. coli* isolates. Among them, the derivative of quinolones, sparfloxacin, demonstrated 100% sensitivity, followed by the sensitivity of carbapenems, imipenem, at 88.23% and the sensitivity of meropenem at 85.29%. All *E. coli* isolates, with a mild to moderate sensitivity to ertapenem (67.64%), were highly resistant to aztreonam and nitrofurantoin (58.82%), nalidixic acid (52.94%), and norfloxacin (41.17%), respectively. The results showed that the antibiotics sparfloxacin and meropenem were the most effective against these isolates. When these bacterial isolates were present not only in clinical lines but also in environmental circumstances, the resistance level of *E. coli* rapidly increased, as shown in Table 1 (Supplementary Data: Annex 1).

The proportion of sensitivity/resistance of several antibiotic classes against *Salmonella spp*. was determined and recorded. Antibiotic sensitivity was documented with antibiotics with meropenem (carbapenems) (86.66%) and (aminoglycosides) tobramycin (86.66%) against all *Salmonella* spp. isolates. Most *Salmonella* spp. isolates demonstrated mild to moderate sensitivity to ertapenem (11) 73.33%, co-trimoxazole (10) 66.66%, and chloramphenicol (9) 60.0%, respectively. Nitrofurantoin (8) 53.33%, ofloxacin (8) 53.33%, and piperacillin (7) 46.66% were shown to be the most resistant. The findings demonstrated that *Salmonella* spp. isolates were nearly resistant to three types of antibiotics. According to the current findings, the antibiotic classes meropenem and tobramycin are the best choices for *Salmonella* spp. isolates, as shown in Table 2 (Supplementary Data: Annex 2).

The proportion of sensitivity/resistance of several antibiotic classes against *Shigella* spp. was determined and recorded. Six classes of antibiotics (8 antibiotics) were accepted against the 32 isolates of *Shigella* spp., none showing 100% sensitivity except meropenem (82.15%), nitrofurantoin (75.0%), gentamycin (62.5%), ertapenem (59.37%), and piperacillin (59.37%). Of eight antibiotics, three classes of antibiotics, co-trimoxazole (56.25%), ofloxacin (46.87%), and tobramycin (46.87%), were resistant to *Shigella* spp. isolates. Meropenem and nitrofurantoin are the best choices against these isolates. The findings demonstrated that, on occasion, cross-resistance between humans and the environment also plays a significant influence in the development of resistance in microorganisms, as shown in Table 3 (Supplementary Data: Annex 3).

Table 4 shows the sensitivity/resistance percentages of various antibiotic classes. Four classes of antibiotics were used against the 23 isolates of *Staphylococcus aureus*, among those of the three antibiotics of quinolones, mild moderately sensitive sparfloxacin (21) 91.30%, moxifloxacin (20) 86.95%, and lomefloxacin (19) 82.60%, the class of glycopeptide antibiotic vancomycin (20) 86.95%, the class of rifamycin, rifamycin (17) 73.91%, the class of glycopeptide, such as vancomycin showed the class of antibiotic quinolones (moxifloxacin). Ampicillin (12) 52.17%, amoxicillin/culvanate (13) 56.52%, and methicillin (5) 21.73% (*β*-lactam antibiotic class) were shown to be highly resistant against isolates of *Staphylococcus aureus.* Sparfloxacin, vancomycin, and moxifloxacin were found to be the best drugs of choice for a large number of *Staphylococcus aureus* isolates. Further classes of B-lactam antibiotics were shown to be extremely resistant, as shown in Table 4 (Supplementary Data: Annex 4).

The sensitivity/resistance proportion of several antibiotic classes against *Pseudomonas aeruginosa* was determined and documented. Five classes of antibiotics were used against isolates of *Pseudomonas aeruginosa*, with the Aminoglycosides family of antibiotics showing the highest sensitivity, such as tobramycin (21) 100%, gentamycin (19) 90.47%, and piperacillin (17) 80.95%, (*β*-lactam antibiotic/aminopenicillins). Mild resistant isolates of *Pseudomonas aeruginosa* with norfloxacin (16) 76.19%, ofloxacin (13) 61.90%. Highly resistant to the monobactam and aminoglycoside class of antibiotics, amikacin (10) exhibited 47.61% resistance and aztreonam (9) exhibited 42.85% resistance. Regarding this, tobramycin and gentamycin were the most effective antibiotics against numerous isolates of *Pseudomonas aeruginosa*. The buffer zone results were given to some types of antibiotics, and the results were close to the resistant zones as shown in Table 5 (Supplementary Data: Annex 5).

The percentage of sensitivity/resistance of various antibiotic classes against *Proteus* spp. was calculated and recorded in Table 6. Six antibiotic classes were used against 11 isolates of *Proteus* spp., with 100% aminoglycoside sensitivity observed for tobramycin (11) and nitrofuran (nitrofurantoin 2) (80.81%), sulfonamides (co-trimoxazole 8) (77.22%), and gentamycin 7 (63.63%). It was interesting to note that among the four various isolates of the present research, *Proteus* spp. was documented to have the highest resistance level, such as piperacillin (45.45%), meropenem (36.36%), ertapenem (27.27%), and norfloxacin (18.18%). Tobramycin and nitrofurantoin were the most effective against many isolates of *Proteus* spp. This finding revealed that the number of antibiotic classes actively involved in inhibition is proportional to the number of antibiotics that develop resistance, as shown in Table 6 (Supplementary Data: Annex 6).

The multiple antibiotic resistance index (MAR) was calculated for all isolates of six genus (Table 7). It was observed that all strains isolated from wastewater treatment plant samples had a MAR index value of more than 0.2. This clearly indicated that all strains might have originated from high-risk sources of contamination. Multiple antibiotic resistance indices were calculated for all multidrug-resistant isolates. Among the 52 isolates of multiple drug resistance bacteria, *E. coli* and *Shigella* spp. had index values ranging from 0.37 to 0.75, *Salmonella* spp. and *Staphylococcus aureus* had MAR index values ranging from 0.5 to 0.75, and *Pseudomonas aeruginosa* and *Proteus* spp. had MAR values ranging from 0.5 to 0.62. Among the six genes, bacterial isolates with multiple antibiotic resistances showed that *Salmonella* spp., *Shigella* spp., and *Staphylococcus aureus* had the highest score values. Although wastewater treatment samples contained coliforms and some pathogenic bacteria organisms, the presence of these organisms indicates that the wastewater samples contain coliforms and some pathogenic bacteria organisms, which can lead to waterborne diseases, as shown in Table 7.

## 4. Discussion

Antimicrobial resistance (AMR) has resulted in increased morbidity and mortality as a result of treatment failures, as well as increased health care costs. Although determining the exact public health risk and estimating the cost increase is difficult, there is little doubt that antibiotic resistance is a serious global issue [19].

In this study, 8.94% of *E. coli* isolates were resistant to four antibiotic classes; the prevalence of *E. coli* isolates in the wastewater treatment plant and the sensitivity/resistance of various antibiotic classes against *Escherichia coli* were assessed and recorded in Table 1. 34 *E. coli* isolates were treated with eight antibiotics from four different classes. Quinolone derivatives, such as sparfloxacin, showed 100% sensitivity, followed by carbapenems such as imipenem, which showed 88.23% sensitivity, and meropenem, which showed 85.29% sensitivity. All *E. coli* isolates had moderate resistance to ertapenem (67.64%), nitrofurantoin (58.82%), aztreonam (58.82%), nalidixic acid (52.94%), and norfloxacin (41.77%). Sparfloxacin and meropenem antibiotics were found to be the most effective against these isolates. *Salmonella* spp. 15 (3.94%) and *Shigella* spp. 32 (8.42%) were documented based on the percentage of sensitivity or resistance of various antibiotic classes against *Shigella* spp. evaluated and recorded in Table 3. Six classes of antibiotics were admitted for the 32 isolates of *Shigella* spp. Among those, none of the classes of antibiotics showed 100 sensitivity, rather than the nitrofurantoin (75.00%), meropenem (81.25%), piperacillin (59.37%), and gentamycin (62.5%) that were documented, respectively. Almost five classes of antibiotics, ertapenem (59.37%), co-trimoxazole 18 (56.25%), ofloxacin (46.87%), and tobramycin (46.87%), were resistant to *Shigella* spp. isolates. Of the 32 *Shigella* spp. isolates, 17 isolates were recorded as MDR in six classes of antibiotics such as carbapenems, quinolones, sulfonamides, B-lactams/aminopenicillin, nitrofuran, and aminoglycosides. The value of the 0.37 to 0.75 MAR index was recorded against *Shigella* spp. isolates.

A total of six antibiotic classes (8 drugs) were tested against all *Salmonella* spp. isolates, although none of the classes showed 100% sensitivity. Tobramycin (aminoglycoside class) and 86.66% were followed by meropenem (carbapenem class) and 86.66%, respectively. *Salmonella* spp. isolates were moderately suppressed by ertapenem (73.33%), co-trimoxazole (sulfonamide class) at 66.66%, and chloramphenicol at 60%. Antibiotics from nearly three classes were resistant to *Salmonella* isolates, including loxacin and nitrofurantoin (53.53%) and piperacillin (46.66%). This is due to repeated exposure to antibiotics and the widespread presence of *Salmonella* spp. in the environment. *Salmonella* spp., isolates from wastewater and surface waters contaminated with sewage, were resistant to one or more chemicals, according to Alcaide and Garay [20]. Streptomycin resistance was the most frequent, followed by ampicillin resistance, with 36.6% resistant to one and 63.4% resistant to two or more antibiotics. Pignato et al. [21] found that only one (2.8%) of 36 enteric serovars from *S. enteric typhimurium* isolates from municipal wastewater were sensitive to all antibiotics tested, while 35 (97.2%) were resistant to one or more antibiotics, including 33 (91.7%) resistant to ampicillin and 18 (50%) were resistant to six antibiotics (ampicillin, chloramphenicol, sulfamethoxazole, tetracycline, streptomycin, and kanamycin).

In Tunisia, studies revealed a large number of pathogenic bacteria, such as *Salmonella* spp., that have a serious influence on human health [22]. *Salmonella* spp. have the highest incidence because they are strongly linked to typhoid fever, paratyphoid fever, and gastroenteritis, especially in developing nations. Only 5 strains of *Staphylococcus aureus* were MDR and sensitive to sparfloxacin, moxifloxacin, lomefloxacin, and vancomycin among the total of 23 culture-positive isolates. According to data from the master sheet on antibiotic patterns of *Staphylococcus aureus,* classes of B-lactam antibiotics were extremely resistant. The prevalence rates of MDR in the current study were 5/23 (21.7%), and this result of the prevalence of MDR was recognized compared to other research by scholars from other countries. The rate of prevalence could also vary from trial to trial, from country to country, or from one area to another within a country, as Padungtod and Kaneene [23] explained. Emmanuel et al. [24] examined the prevalence of various antibiotic-resistant strains of *P. aeruginosa* at three wastewater treatment plants (Alice, Dimbaza, and East London). They discovered that the isolates studied were exceptionally sensitive to gentamycin (100%), ofloxacin (100%), and penicillin (100%), followed by clindamycin (90%), erythromycin (90%), rifampin (90%), sulfamethoxazole (90%), nitrofurantoin (80%), and cephems (70%). There were 5 MDR strains among the 21 culture-positive isolates of *Pseudomonas aeruginosa*. These strains have 100% sensitivity. Aminoglycoside antibiotics (Tobramycin) and others with medium to moderate sensitivity, such as gentamycin, piperacillin, imipenem, and norfloxacin, were very resistant, as were ofloxacin, amikacin, and aztreonam. The current prevalence rates for *Pseudomonas aeruginosa* MDR were 5/21 (23.80%), which was lower than previous studies conducted in Abidjan by Benie et al. (2016), which documented a prevalence of *Pseudomonas aeruginosa* of 29.7%, which was higher than this study (23.8%). The study carried out at Al-Shifa Hospital in Gaza compared the contribution of hospital wastewater to the spread of antibiotic resistance in nonhealth institutions. The bacterium most frequently identified was *Pseudomonas* spp. (33.1%), followed by *E. coli* (30.5%), *Enterococcus* spp. (21.4%), *Klebsiella* spp. (10.4%), and *Proteus* spp. (4.5%). In this study were *Proteus* spp. 11 (2.89), were isolated from a wastewater treatment plant. Compared to the previous study, the occurrence of *Proteus spp.* was lower because the wastewater treatment plant reduced the percentage of sensitivity/resistance of various classes of antibiotics against *Proteus* spp., as shown in Table 6. Six antibiotic classes were used to treat 11 isolates of *Proteus* spp. Among the 100% sensitivity recorded with tobramycin and moderate sensitivity nitrofurantoin, 81.81% with co-trimoxazole 72.72% and gentamycin, 63.63% were documented, respectively. Three classes of antibiotics showed resistant piperacillin 45.45%, meropenem, 36.36%, ertapenem 27.27% and (norfloxacin), 18.18%, respectively. This result revealed how few classes of antibiotics are actively involved in the inhibition of antibiotics and how many antibiotics undergo resistance.

Table 7 shows the prevalence of MDR bacterial isolates in wastewater treatment plant samples. Bacterial isolates that are resistant to three or more classes of antibiotics were classified as multidrug-resistant (MDR) isolates according to the result of sensitivity/resistance. Thirteen *E. coli* isolates were identified as MDR, and four antibiotic classes were resistant to these isolates. Out of 15 *Salmonella* spp. isolates, 06 were recorded as MDR, with seven classes of antibiotics included; out of 32 *Shigella* spp. isolates, 17 isolates were documented as MDR and resistant to five classes of antibiotics; of the 23 *Staphylococcus aureus* isolates 05 isolates were documented as MDR; and out of *Pseudomonas* spp., isolates, the maximum of isolates were resistant to five classes of antibiotics 05 was observed as MDR and resistant with of 11 *Proteus* spp. isolates, 06 were observed as MDR and maximum of resistant with six classes of antibiotics. Although wastewater samples were found to contain coliforms as well as some pathogenic organisms, the existence of these organisms indicates that wastewater treatment plant samples had coliforms as well as some pathogenic organisms, resulting in waterborne diseases. Treatment of hospital wastewater may not be completely efficient in eradicating multiple drug-resistant bacteria and resistant genes from hospital wastewater, according to studies conducted in Australia [25], Brazil [26], and China [27]. When MDR bacteria are released into the environment, they promote widespread genetic exchange, and opportunistic diseases (often found in free-living populations) can become resistant as a result of the resistance mechanisms they acquire. As a result, reducing selective pressure by restricting antibiotic use is a critical step in stopping the spread of resistance in hospital wastewater and ensuring that resistant organisms are not favored [28].

For each of the six genera, the multiple antibiotic index (MAR) was determined (Table 7). The MAR index value of the strains isolated from samples from wastewater treatment facilities was found to be greater than 0.2. This clearly demonstrated that the strains could have come from sources of high-risk contamination. According to the findings of this study, the resistance of these organisms is defined only by their resistance levels to three or more antibiotic classes. *E. coli* and *Shigella* spp. had index values ranging from 0.37 to 0.75, *Salmonella* spp. and *Staphylococcus aureus* had MAR index values ranging from 0.5 to 0.75, and *Pseudomonas aeruginosa* and *Proteus* spp. had MAR values ranging from 0.5 to 0.62.

## 5. Conclusions

The results presented indicate that wastewater treatment plants are an important source of antibiotic-resistant bacteria. Some organisms were sensitive to sparfloxacin and tobramycin, while *Staphylococcus aureus* was sensitive to methicillin and others showed the highest resistance. The contamination of wastewater treatment by antibiotics or other pollutants leads to the rise of resistance due to selection pressure. The presence of antibiotic-resistant organisms in this waste water should not be overlooked. There must be an appropriate wastewater treatment and disposal system at the university. Changing the treatment technology or treatment plant to produce good-quality effluent suitable for disposal into the environment is required; there should be proper design and implementation of waste stabilization ponds as anaerobic, facultative, and maturation ponds in series that are economical for a wastewater treatment system. This indicates the need to improve the new modifications of wastewater treatment processes and the continuous monitoring of treated sewage discharged into the environment.

## Figures and Tables

**Table 1:** Antimicrobial susceptibility profiles of *E. coli* at the Wolaita Sodo University wastewater treatment plant.

Antibacterial agents and its strength (mcg)	Symbol	*Escherichia coli* (*n* = 34)		
		*S* (%)	*I* (%)	*R* (%)
Imipenem (10)	IMP	30 (88.23)	4 (11.76)	—
Meropenem (10)	MRP	29 (85.29)	—	05 (14.70)
Ertapenem (10)	ETP	23 (67.64)	—	11 (32.35)
Aztreonam (30)	AT	20 (58.82)	01 (2.94)	13 (38.23)
Nalidixic acid (30)	NA	18 (52.94)	—	16 (47.05)
Sparfloxacin (5)	SPX	34 (100)	—	—
Norfloxacin (10)	NX	14 (41.17)	—	20 (58.82)
Nitrofurantoin (300)	NIT	20 (58.82)	—	14 (41.17)

*S*, sensitivity; *I*, intermediate; *R*, resistant.

**Table 2:** Antimicrobial susceptibility profiles of *Salmonella spp*. at the Wolaita Sodo University wastewater treatment plant.

Antibacterial agents and its strength (mcg)	Symbol	*Salmonella spp*. (*n* = 15)		
		*S* (%)	*I* (%)	*R* (%)
Meropenem (10)	MRP	13 (86.66)	—	2 (13.33)
Ertapenem (10)	ETP	11 (73.33)	—	4 (26.66)
Ofloxacin (5)	OF	8 (53.33)	01 (6.66)	4 (40)
Co-trimoxazole (125)	COT	10 (66.66)	—	5 (33.33)
Piperacillin (30)	PI	7 (46.66)	—	8 (53.33)
Chloramphenicol (30)	C	9 (60)	—	6 (40)
Tobramycin (10)	TOB	13 (86.66)	—	2 (13.33)
Nitrofurantoin (300)	NIT	8 (53.33.82)	—	7 (46.66)

*S*, sensitivity; *I*, intermediate; *R*, resistant.

**Table 3:** Antimicrobial susceptibility profiles of *Shigella spp*. at the Wolaita Sodo University wastewater treatment plant.

Antibacterial agents and its strength (mcg)	Symbol	*Shigella* spp. (*n* = 32)		
		*S* (%)	*I* (%)	*R* (%)
Meropenem (10)	MPR	26 (81.25)	—	6 (18.75)
Ertapenem (10)	ETP	19 (59.37)	—	13 (40.62)
Ofloxacin (5)	OF	15 (46.87)	02 (6.25)	15 (46.87)
Co-trimoxazole (125)	COT	18 (56.25)	—	14 (43.75)
Piperacillin (30)	PI	19 (59.37)	03 (9.37)	10 (31.25)
Tobramycin (10)	TOB	15 (46.87)	—	17 (53.12)
Gentamycin (10)	GEN	20 (62.5)	—	12 (37.5)
Nitrofurantoin (300)	NIT	24 (75.00)	—	08 (25.00)

*S*, sensitivity; *I*, intermediate; *R*, resistant.

**Table 4:** Antimicrobial susceptibility profiles of *Staphylococcus aureus* at the Wolaita Sodo University wastewater treatment plant.

Antibacterial agents and its strength (mcg)	Symbol	*Staphylococcus aureus* (*n* = 32)		
		*S* (%)	*I* (%)	*R* (%)
Moxifloxacin (5)	MO	20 (86.95)	—	3 (13.04)
Sparfloxacin (5)	SPX	21 (91.30)	—	2 (8.69)
Lomefloxacin (10)	LOM	19 (82.60)	—	4 (17.39)
Rifamycin (5)	RIP	17 (73.91)	—	6 (26.08)
Amoxicillin-cluvanate (30)	AMC	13 (56.52)	—	10 (43.4)
Ampicillin (10)	AMP	12 (52.17)	—	11 (47.82)
Methicillin (5)	MET	5 (21.73)	—	18 (78.26)
Vancomycin (5)	VA	20 (86.95)	—	3 (13.04)

*S*, sensitivity; *I*, intermediate; *R*, resistant.

**Table 5:** Antimicrobial susceptibility profiles of *Pseudomonas aeruginosa* at the Wolaita Sodo University wastewater treatment plant.

Antibacterial agents and its strength (mcg)	Symbol	*Pseudomonas aeruginosa* (*n* = 21)		
		*S* (%)	*I* (%)	*R* (%)
Imipenem (10)	IPM	15 (71.42)	2 (9.52)	4 (19.04)
Ofloxacin (5)	OF	13 (59.37)	3 (14.28)	5 (23.80)
Norfloxacin (10)	NX	16 (76.19)	—	5 (23.80)
Aztreonam (30)	AT	9 (42.85)	—	12 (57.14)
Piperacillin (30)	PI	17 (80.95)	1 (4.76)	3 (14.28)
Tobramycin (10)	TOB	21 (100)	—	—
Gentamycin (10)	GEN	19 (90.47)	—	2 (9.52)
Amikacin (30)	AK	10 (47.61)	—	11 (52.38)

*S*, sensitivity; *I*, intermediate; *R*, resistant.

**Table 6:** Antimicrobial susceptibility profiles of *Proteus* spp. at the Wolaita Sodo University wastewater treatment plant.

Antibacterial agents and its strength (mcg)	Symbol	*Proteus* spp. (*n* = 11)		
		*S* (%)	*I* (%)	*R* (%)
Meropenem (10)	MPR	4 (36.36)	—	7 (67.67)
Ertapenem (10)	ETP	3 (27.27)	—	8 (72.72)
Norfloxacin (5)	NX	2 (18.18)	—	9 (81.81)
Co-trimoxazole (125)	COT	8 (72.72)	—	3 (27.27)
Piperacillin (30)	PI	5 (45.45)	—	6 (54.54)
Tobramycin (10)	TOB	11 (100)	—	—
Gentamycin (10)	GEN	7 (62.5)	—	4 (36.36)
Nitrofurantoin (300)	NIT	9 (81.81)	2 (18.18)	—

*S*, sensitivity; *I*, intermediate; *R*, resistant.

**Table 7:** Multiple antibiotic-resistant (MAR) index values of resistant isolates.

S. no.	MAR index value	No. of strains	Percentage
*Escherichia coli*			
1	0.37	2	15.38
2	0.5	9	69.23
3	0.62	2	15.38
Total		**13**	**100**

*Salmonella* spp.			
1	0.5	2	33.33
2	0.62	3	50.00
3	0.75	1	16.66
Total		**6**	**100**

*Shigella* spp.			
1	0.37	3	17.65
2	0.5	4	23.53
3	0.62	8	47.06
4	0.75	2	11.76
Total		**17**	**100**

*Staphylococcus aureus*			
1	0.5	1	20
2	0.62	3	60
3	0.75	1	20
Total		**5**	**100**

*Pseudomonas aeruginosa*			
1	0.5	4	80
2	0.62	1	20
Total		**5**	**100**

*Proteus* spp.			
1	0.5	4	66.67
2	0.62	2	33.33
Total		**6**	**100**

## Data Availability

The data used to support the findings of this study are available from the corresponding author upon request.

## Abbreviations

MDR: Multiple drug resistance
MAR: Multiple antibiotic-resistant
AST: Antibiotic susceptible test
ARB: Antibiotic-resistant bacteria
ARG: Antibiotic resistance genes
WWTPs: Waste water treatment plants.

## Supplementary Materials

Supplementary Materials The supplementary data based on each sensitivity/resistant patterns in the isolated bacterial species are reported (Annex 1–6).
