# Supplementary material for: Prevalence of Multiple Drug-Resistant Bacteria in the Main Campus Wastewater Treatment Plant of Wolaita Sodo University, Southern Ethiopia
Source: Int J Microbiol. 2022 Nov 23;2022:1781518. doi: 10.1155/2022/1781518 (PMC9711955; doi:10.1155/2022/1781518)
Supplement: Supplementary Materials — The supplementary data based on each sensitivity/resistant patterns in the isolated bacterial species are reported (Annex 1–6). [file 1781518.f1.docx]

**Supplementary data**

**Annex 1: Sensitivity/Resistant patterns of isolated bacteria from wastewater treatment plant samples**

**Sensitivity/Resistant patterns of *Escherichia coli***

| S.No. | Name of the isolate | **Resistant/Sensitivity pattern – Zone of Diameter in mm** | | | | | | | | |
| --- | --- | --- | --- | --- | --- | --- | --- | --- | --- | --- |
|  |  | AT | ETP | IMP | | MRP | NA | SPX | NX | NIT |
| 1 | *E.coli* | R(20) | S(26) | | S(35) | S(23) | R(--) | S(21) | R(14) | S(25) |
| 2 | *E.coli* | S(24) | S(27) | | I(20) | S.(23) | S(20) | S(30) | R(13) | S(21) |
| 3 | *E.coli* | R(18) | S(22) | | S(26) | S(22) | S(23) | S(22) | S(23) | R(10) |
| 4* | *E.coli* | S(28) | R(17) | | S(31) | S(25) | R(06) | S(30) | R(16) | S(21) |
| 5* | *E.coli* | S(24) | R(20) | | S(23) | S(22) | R(06) | S(20) | R(16) | R(--) |
| 6 | *E.coli* | S(25) | S(26) | | S(22) | S(30) | S(21) | S(24) | S(22) | S(20) |
| 7* | *E.coli* | R(16) | R(15) | | S(25) | S(27) | R(--) | S(31) | R(--) | R(--) |
| 8 | *E.coli* | S(29) | S(25) | | S(30) | S(26) | S(24) | S(35) | S(26) | S(22) |
| 9 | *E.coli* | R(22) | S(24) | | I(20) | S(22) | S(21) | S(24) | R(11) | S(20) |
| 10 | *E.coli* | S(28) | S(25) | | S(30) | S(26) | R(09) | S(32) | S(22) | R(--) |
| 11 | *E.coli* | S(29) | S(29) | | S(28) | S(22) | S(25) | S(24) | S(22) | S(28) |
| 12* | *E.coli* | R(18) | R(08) | | S(31) | R(17) | R(--) | S(30) | R(--) | S(19) |
| 13 | *E.coli* | S(32) | S(31) | | S(23) | S(30) | S(22) | S(28) | S(25 | S(29) |
| 14* | *E.coli* | S(24) | R(19) | | S(32) | S(23) | R(07) | S(20) | R(12) | R(--) |
| 15* | *E.coli* | S(26) | R(20) | | S(30) | S(27) | R(12) | S(27) | R(10) | R(--) |
| 16* | *E.coli* | S(30) | R(20) | | S(24) | S(24) | R(11) | S(20) | R(12) | R(--) |
| 17 | *E.coli* | R(22) | S(30) | | S(22) | S(36) | S(20) | S(21) | S(26) | S(27) |
| 18 | *E.coli* | S(34) | S(27) | | S(30) | S(30) | S(22) | S(24) | S(22) | R(15) |
| 19* | *E.coli* | R(21) | R(20) | | I(21) | S(23) | R(08) | S(22) | R(--) | S(22) |
| 20 | *E.coli* | I(23) | S(22) | | S(31) | S(24) | S(22) | S(20) | S(27) | R(--) |
| 21 | *E.coli* | S(26) | R(20) | | S(36) | S(26) | R(--) | S(22) | S(26) | S(25) |
| 22 | *E.coli* | R(21) | S(25) | | I(20) | S(31) | S(30) | S(26) | R(13) | S(28) |
| 23 | *E.coli* | S(37) | S(32) | | S(29) | R(20) | S(24) | S(30) | S(24) | S(27) |
| 24* | *E.coli* | R(20) | S(29) | | S(34) | S(23) | R(11) | S(31) | R(15) | R(--) |
| 25* | *E.coli* | S(30) | R(18) | | S(26) | S(25) | S(25) | S(32) | R(11) | R(--) |
| 26 | *E.coli* | R(16) | S(30) | | S(23) | S(24) | S(26) | S(32) | S(28) | S(19) |
| 27 | *E.coli* | S(36) | S(36) | | S(28) | S(32) | S(26) | S(31) | R(09) | S(18) |
| 28* | *E.coli* | R(21) | S(27) | | S(22) | R(20) | S(23) | S(31) | R(12) | R(08) |
| 29* | *E.coli* | R(22) | S29) | | S(30) | S(26) | R(--) | S(29) | R(--) | R(--) |
| 30* | *E.coli* | S(36) | S(28) | | S(28) | S(24) | S(21) | S(31) | S(27) | S(18) |
| 31* | *E.coli* | R(14) | S(22) | | S(24) | R(12) | R(13) | S(28) | R(14) | S(17) |
| 32* | *E.coli* | S(31) | R(13) | | S(28) | S(24) | S(28) | S(26) | R(10) | R(--) |
| 33 | *E.coli* | S(24) | S(29) | | S(31) | S(23) | R(10) | S(28) | S(26) | S(28) |
| 34 | *E.coli* | S(25 | S(27) | | S(28) | R(20) | R(11) | S(30) | R(12) | S(18) |

**(S)**-Sensitivity, (**I)** - Intermediate, (**R)** – Resistant, (--) **-** NoZone also resistant

**Annex 2: Sensitivity/Resistant patterns of *Salmonella* spp.**

| **S.No.** | **Name of the isolate** | **Resistant/Sensitivity pattern – Zone of Diameter in mm** | | | | | | | | | | |
| --- | --- | --- | --- | --- | --- | --- | --- | --- | --- | --- | --- | --- |
|  |  | C | COT | | MRP | ETP | | OF | | PI | TOB | NIT |
| 1 | *Salmonella*  spp. | R(--) | | S(22) | S(26) | | S(29) | | S(25) | S(20) | S(21) | S(27) |
| 2 | *Salmonella*  spp. | S(19) | | R(15) | S(36) | | S(33) | | R(11) | S(26) | S(28) | S(20) |
| 3 | *Salmonella*  spp. | R(--) | | S(18) | S(29) | | S(25) | | S(24) | R(16) | S(22) | S(17) |
| 4* | *Salmonella*  spp. | R (9) | | S(21) | R(08) | | S(26) | | R(12) | R(12) | S(26) | R(--) |
| 5* | *Salmonella*  spp. | S(19) | | R(14) | S(30) | | R(--) | | S(35) | R(14) | S(23) | R(--) |
| 6 | *Salmonella*  spp. | S(30) | | S(28) | S(36) | | S(34) | | S(34) | R(10) | S(25) | S(24) |
| 7 | *Salmonella*  spp. | S(20) | | S(32) | S(36) | | R(--) | | S(30) | S(21) | R(--) | S(20) |
| 8 | *Salmonella*  spp. | S(18) | | S(22) | S(26) | | S(25) | | I(20) | R(13) | S(24) | R(--) |
| 9* | *Salmonella*  spp. | R(--) | | R(10) | S(26) | | S(24) | | R(15) | R(11) | S(22) | R(--) |
| 10* | *Salmonella*  spp. | R(--) | | R(13) | S(28) | | R(10) | | R(10) | R(14) | S(22) | R(10) |
| 11 | *Salmonella*  spp. | S(24) | | S(28) | S(26) | | S(23) | | S(31) | S(24) | S(26) | S(23) |
| 12 | *Salmonella*  spp. | S(20) | | R(11) | S(26) | | S(24) | | S(22) | S(25) | S(22) | S(19) |
| 13* | *Salmonella*  spp. | R(08) | | S(16) | R(--) | | S(22) | | R(18) | S(25) | S(29) | R(--) |
| 14* | *Salmonella*  spp. | S(18) | | S(16) | S(23) | | R(11) | | R(08) | R(10) | R(--) | R(--) |
| 15 | *Salmonella*  spp. | S(21) | | S(17) | S(31) | | S(24) | | S(28) | S(26) | S(34) | S(26) |

**(S)**-Sensitivity, (**I)** - Intermediate, (**R)** – Resistant, (--) **–** No Zone also resistant

**Annex 3: Senstivity/Resistant patterns of *Shigella* spp.**

| **S.No.** | **Name of the isolate** | **Resistant/Sensitivity pattern – Zone of Diameter in mm** | | | | | | | | | |
| --- | --- | --- | --- | --- | --- | --- | --- | --- | --- | --- | --- |
|  |  | GEN | TOB | COT | MRP | | ET P | | PI | NIT | OF |
| 1 | *Shigella* spp. | S(23) | S(25) | S(19) | | S(28) | | S(23) | S(28) | R(--) | S(24) |
| 2* | *Shigella* spp. | S(28) | R(--) | R(10) | | S(34) | | R(11) | S(22) | S(18) | S(29) |
| 3 | *Shigella* spp. | R(09) | S(31) | S(31) | | S(29) | | S(29) | S(22) | S(17) | S(26) |
| 4* | *Shigella* spp. | S(22) | R(11) | R(--) | | S(25) | | R(13) | R(17) | S(20) | S(22) |
| 5* | *Shigella* spp. | R(--) | R(13) | R(8) | | R(--) | | S(25) | S(28) | S(21) | R(18) |
| 6* | *Shigella* spp. | S(17) | R(--) | S(22) | | S(25) | | R(14) | R(16) | S(17) | S(22) |
| 7 | *Shigella* spp. | S(22) | S(22) | S(22) | | S(29) | | S(29) | R(17) | S(19) | S(28) |
| 8 | *Shigella* spp. | R(08) | S(24) | S(25) | | S(23) | | R(16) | S(21) | S(20) | I (21) |
| 9* | *Shigella* spp. | S(22) | R(13) | R(--) | | R(--) | | S(29) | R(--) | S(18) | R(11) |
| 10 | *Shigella* spp. | S(24) | S(26) | S(24) | | R(11) | | S(28) | S(23) | R(--) | S(22) |
| 11* | *Shigella* spp. | R(--) | R(12) | R(--) | | S(28) | | R(11) | S(22) | S(22) | R(18) |
| 12 | *Shigella* spp. | S(24) | S(29) | S(17) | | S(27) | | S(26) | S(21) | R(--) | S(23) |
| 13* | *Shigella* spp. | R(--) | R(10) | R(--) | | S(27) | | R(14) | S(26) | S(17)) | R(16) |
| 14 | *Shigella* spp. | S(23) | S(22) | S(28) | | S(30) | | S(29) | S(23) | R(--) | S(22) |
| 15 | *Shigella* spp. | S(22) | R(--) | S(20) | | S(29) | | S(28) | I(19) | S(17) | S(22) |
| 16* | *Shigella* spp. | R(--) | R(--) | S(17) | | S(31) | | R(--) | R(11) | S(18) | R(12) |
| 17 | *Shigella* spp. | S(24) | S(21) | S(23) | | S(32) | | R(9) | S(20) | S(19) | S(22) |
| 18 | *Shigella* spp. | S(23) | S(30) | S(19) | | S(30) | | R(--) | I(19) | S(19) | R(15) |
| 19* | *Shigella* spp. | R(--) | R(--) | R(08) | | S(24) | | S(27) | R(16) | R(--) | R(14) |
| 20* | *Shigella* spp. | R(--) | R(14) | R(12) | | S(22) | | R(11) | S(22) | S(18) | S(22) |
| 21 | *Shigella* spp. | S(26) | S(29) | S(18) | | S(24) | | S(23) | S(21) | S(20) | R(19) |
| 22* | *Shigella* spp. | S(20) | S(24) | R(10) | | S(27) | | R(--) | S(21) | S(19) | R(16) |
| 23* | *Shigella* spp. | R(--) | R(12) | S(18) | | R(--) | | S(24) | S(20) | R(08) | R(--) |
| 24 | *Shigella* spp. | S(24) | S(24) | S(19) | | S(31) | | S(26) | S(21) | S(20) | R(16) |
| 25* | *Shigella* spp. | R(09) | R(11) | R(--) | | S(29) | | S(30) | S(23) | S(17) | R(11) |
| 26* | *Shigella* spp. | R(--) | R(--) | R(10) | | R(--) | | S(23) | R(15) | S(19) | S(22) |
| 27 | *Shigella* spp. | S(23) | S(20) | S(18) | | S(26) | | S22) | S(21) | S(20) | R(12) |
| 28* | *Shigella* spp. | S(23) | R(11) | R(11) | | S(24) | | R(14) | R(16) | R(--) | R(16) |
| 29 | *Shigella* spp. | S(20) | S(26) | S(27) | | S(28) | | S(24) | S(20) | S(20) | I(21) |
| 30 | *Shigella* spp. | S(21) | R(--) | S(19) | | S(29) | | S(31) | I(19) | R(12) | S(22) |
| 31* | *Shigella* spp. | R(--) | S(28) | R(9) | | R(--) | | R(--) | R(14) | S(19) | S(23) |
| 32* | *Shigella* spp. | S(25) | R(11) | R(--) | | S(32) | | S(23) | R(11) | S(21) | R(15) |

**(S)**- Sensitivity, (**I)** - Intermediate, (**R)** – Resistant, (--) **-** No Zone also resistant

**Annex 4: Sensitivity/Resistant patterns of *Staphylococcus* *aureus***

| S.No. | Name of the isolate | **Resistant/Sensitivity pattern – Zone of Diameter in mm** | | | | | | | | |
| --- | --- | --- | --- | --- | --- | --- | --- | --- | --- | --- |
|  |  | AMC | AMP | MET | MO | SPX | LOM | | VA | RIF |
| 1 | *Staph aureus* | S(20) | R(18) | R(--) | S(25) | S(22) | | S(27) | R(18) | S(30) |
| 2 | *Staph*  *aureus* | S(20) | S(31) | S(22) | S(29) | S(20) | | S(25) | S(23) | S(28) |
| 3 | *Staph aureus* | S(20) | R(--) | R (08) | R(22) | R(16) | | S(26) | S(21) | R(18) |
| 4 | *Staph aureus* | R(18) | S(32) | R(08) | S(28) | S(19) | | S(23) | S(21) | S(32) |
| 5 | *Staph aureus* | S(20) | R(17) | R(--) | R(21) | S(27) | | R(14) | R(16) | S(26) |
| 6 | *Staph aureus* | S(20) | S(31) | R(09) | S(25) | S(24) | | S(20) | S(26) | R(15) |
| 7 | *Staph aureus* | S(20) | R(19) | S(17) | S(25) | S(20) | | R(--) | S(24) | S(26) |
| 8 | *Staph aureus* | R(--) | S(32) | R(--) | S(24) | S(23) | | S(20) | S(22) | S(26) |
| 9 | *Staph aureus* | R(15) | S(33) | R(09) | S(25) | S(20) | | S(20) | S(29) | R(13) |
| 10 | *Staph aureus* | R(16) | S(30) | R(--) | S(25) | S(19) | | R(17) | S(23) | S(27) |
| 11 | *Staph aureus* | R(12) | R(22) | R(--) | S(29) | R(16) | | S(20) | S(26) | R(19) |
| 12 | *Staph aureus* | S(20) | R(18) | R(--) | S(26) | S(21) | | S(22) | S(25) | S(28) |
| 13 | *Staph aureus* | R(13) | R(25) | S(18) | S(25) | S(20) | | S(26) | S(29) | R(22) |
| 14 | *Staph aureus* | S(20) | S(29) | R(09) | S(26) | S(25) | | S(20) | S(25) | S(26) |
| 15 | *Staph aureus* | S(23) | R(14) | S(17) | S(26) | S(23) | | S(24) | S(23) | S(30) |
| 16 | *Staph aureus* | S(26) | S(26) | R(--) | S(27) | S(26) | | S(23) | S(25) | S(27) |
| 17 | *Staph aureus* | R(11) | S(24) | R(09) | S(29) | S(28) | | S(27) | S(24) | S(26) |
| 18 | *Staph aureus* | R(10) | R(08) | R(--) | R(20) | S(25) | | S(26) | R(17) | R(20) |
| 19 | *Staph aureus* | S(24) | S(27) | R(--) | S(26) | S(27) | | S(28) | S(27) | S(29) |
| 20 | *Staph aureus* | S(23) | R(--) | S(20) | S(28) | S(28) | | S(25) | S(29) | S(25) |
| 21 | *Staph aureus* | R(12) | S(27) | R(--) | R(24) | S(25) | | S(25) | S(24) | S(27) |
| 22* | *Staph aureus* | R(09) | R(09) | R(07) | S(25) | S(23) | | R(13) | S(27) | S(25) |
| 23 | *Staph aureus* | S(25) | S(23) | R(--) | S(29) | S(26) | | S(24) | S(25) | S(28) |

**(S)**- Sensitivity, (**I)** - Intermediate, (**R)** – Resistant, (--) **-** No Zone also resistant

**Annex 5: Sensitivity/Resistant patterns of *Pseudomonas aeruginosa***

| **S.No.** | **Name of the isolate** | **Resistant/Sensitivity pattern – Zone of Diameter in mm** | | | | | | | |
| --- | --- | --- | --- | --- | --- | --- | --- | --- | --- |
|  |  | AK | TOB | GEN | OF | NX | IPM | PI | AT |
| 1 | *Pseudomonas aeruginosa* | S(23) | S(21) | S(20) | I(20) | S(20) | S(26) | S(22) | R(10) |
| 2 | *Pseudomonas aeruginosa* | R(11) | S(22) | S(16) | S(22) | S(18) | S(22) | S(21) | S(25) |
| 3 | *Pseudomonas aeruginosa* | S(22) | S(20) | S(20) | S(22) | S(21) | S(25) | S(20) | R(--) |
| 4 | *Pseudomonas aeruginosa* | R(13) | S(21) | S(20) | S(23) | S(21) | S(28) | S(23) | R(15) |
| 5* | *Pseudomonas aeruginosa* | R(11) | S(24) | S(24) | R(19) | S(19) | R(14) | S(21) | R(--) |
| 6 | *Pseudomonas aeruginosa* | S(18) | S(21) | S(15) | R(12) | R(16) | S(31) | S(24) | R(--) |
| 7 | *Pseudomonas aeruginosa* | S(23) | S(22) | S(24) | S(22) | S(21) | I(18) | I(18) | R(19) |
| 8 | *Pseudomonas aeruginosa* | R(10) | S(26) | S(24) | S(22) | S(20) | S(34) | S(23) | S(29) |
| 9* | *Pseudomonas aeruginosa* | R(12) | S(27) | R(12) | I(20) | R(10) | S(27) | R(15) | R(20) |
| 10 | *Pseudomonas aeruginosa* | S20) | S(22) | S(24) | S(23) | S(24) | R(13) | S(22) | R(10) |
| 11* | *Pseudomonas aeruginosa* | R(11) | S(22) | R(11) | S(22) | R(12) | S(24) | R(17) | S(29) |
| 12 | *Pseudomonas aeruginosa* | S(23) | S(21) | S(25) | R(14) | S(24) | S(23) | S(21) | S(24) |
| 13 | *Pseudomonas aeruginosa* | R(13) | S(21) | S(21) | R(15) | S(22) | S(32) | S(26) | S(27) |
| 14 | *Pseudomonas aeruginosa* | S(20) | S(23) | S(24) | S(24) | S(25) | R(14) | S(22) | S(24) |
| 15 | *Pseudomonas aeruginosa* | R(13) | S(28) | S(21) | S(22) | S(23) | I(17) | S(26) | S(30) |
| 16 | *Pseudomonas aeruginosa* | S(24) | S(20) | S(25) | S(22) | S(20) | S(24) | S(21) | R(08) |
| 17* | *Pseudomonas aeruginosa* | R(11) | S(26) | S(16) | I(20) | R(12) | R(12) | R(15) | S(29) |
| 18 | *Pseudomonas aeruginosa* | S(24) | S(24) | S(24) | S(22) | S(20) | S(26) | S(21) | S(29) |
| 19 | *Pseudomonas aeruginosa* | S(25) | S(24) | S(25) | S(22) | S(21) | S(26) | S(23) | R(--) |
| 20* | *Pseudomonas aeruginosa* | R(13) | S(23) | S(17) | R(14) | R(11) | S(25) | S(22) | R(09) |
| 21 | *Pseudomonas aeruginosa* | R(14) | S(21) | S(16) | S(22) | S(21) | S(22) | S(20) | R(13) |

**(S)**-Sensitivity, (**I)** - Intermediate, (**R)** – Resistant, (--) **–** No Zone also resistant

**Annex 6** - **Sensitivity/Resistant patterns of *Proteus* spp.**

| **S.No.** | **Name of the isolate** | **Resistant/Sensitivity pattern – Zone of Diameter in mm** | | | | | | | |
| --- | --- | --- | --- | --- | --- | --- | --- | --- | --- |
|  |  | **MRP** | **ETP** | **NX** | **COT** | **PI** | **TOB** | **GEN** | **NIT** |
| 1* | *Proteus* spp. | R(09) | R(13) | R(12) | S(25) | R(--) | S(25) | S( 24) | S(26) |
| 2 | *Proteus* spp. | S(24) | R(08) | R(11) | S(24) | S(25) | S(24) | S(26) | S(24) |
| 3* | *Proteus* spp. | R(12) | S(26) | R(--) | S(28) | R( 11) | S(26) | R(13) | S(23) |
| 4 | *Proteus* spp. | R(10) | R(09) | S(28) | R(10) | S(27) | S(26) | S(27) | S(21) |
| 5* | *Proteus* spp. | S(26) | R(--) | R(14) | S(27) | R(09) | S(25) | R(12) | I(16) |
| 6 | *Proteus* spp. | R(--) | S(24) | R(13) | S(25) | S(25) | S(23) | S(28) | S(25) |
| 7* | *Proteus* spp. | R(--) | R(11) | R(12) | S(25) | R(--) | S(29) | R(11) | S(25) |
| 8* | *Proteus* spp. | S(26) | R(12) | R(--) | R(12) | R(12) | S(27) | S(27) | S23) |
| 9 | *Proteus* spp. | S(25) | S(25) | R(11) | S(26) | S(25) | S(26) | S(25) | I(16) |
| 10* | *Proteus* spp. | R(12) | R(--) | R(12) | S(24) | R(09) | S(25) | R09) | S(27) |
| 11 | *Proteus* spp. | R(09) | R(12) | S(26) | R(11) | S(27) | S(29) | S(28) | S(23) |

**(S)**-Sensitivity, (**I)** - Intermediate, (**R)** – Resistant, (--) **–** No Zone also resistant
